# hTPO和hNIS共转染肺癌细胞系H460介导放射性碘摄取的研究

**DOI:** 10.3779/j.issn.1009-3419.2010.06.002

**Published:** 2010-06-20

**Authors:** 玮 李, 建 谭, 雷 龙

**Affiliations:** 300052 天津，天津医科大学总医院核医学科 Department of Nuclear Medicine, Tianjin Medical University General Hospital, Tianjin 300052, China

**Keywords:** 钠/碘同向转运体, 甲状腺过氧化物酶, 重组腺病毒, 基因治疗, 放射性核素治疗, Human sodium/iodide symporter, Tyroperoxidase, Recombinant adenovirus, Gene therapy, Radionuclide therapy

## Abstract

**背景与目的:**

肺癌严重危害人类生命和健康，目前国内外肺癌的发病率和死亡率仍在不断上升。尤其是非小细胞肺癌（non-small cell lung cacer, NSCLC）治疗效果多年来一直没有显著提高。本研究旨在将人甲状腺过氧化物酶（*hTPO*）基因及人钠碘转运体（*hNIS*）基因共转入肺癌细胞系后研究其摄碘能力的变化。

**方法:**

克隆，重组，包装并扩增纯化得到重组腺病毒（AdTPO），测定病毒滴度，Western blot检测重组腺病毒的表达。使用脂质体转染法将*hNIS*基因转染入人肺癌细胞系H460中，经G418抗生素筛选获得稳定表达hNIS的细胞系hNIS-H460，为hNIS-H460组；使用重组腺病毒将*hTPO*基因转导入hNIS-H460中，使肺癌细胞系获得*hTPO*基因，为AdTPO-hNIS-H460组；未转入hTPO和hNIS的细胞为对照组（H460）。然后进行三组稳定表达细胞系的体外摄^125^Ⅰ实验。三组间两两比较用*q*检验（*Newman-Keuls*法）。

**结果:**

AdTPO-hNIS-H460细胞、hNIS-H460细胞和H460细胞所摄取^125^Ⅰ分别为（59 637.67±1 281.13）、（48 622.17±2 242.28.）和（1 440.17±372.86）计数·min^-1^。三组间总体具有统计学差异（*P* < 0.01）。AdTPO-hNIS-H460组较对照组（H460）摄^125^Ⅰ能力增高约41倍（*P* < 0.01），hNIS-H460组较对照组（H460）摄碘能力增高约34倍（*P* < 0.01）。AdTPO-hNIS-H460组较hNIS-H460组高约1.2倍（*P* < 0.01）。

**结论:**

将*hTPO*和*hNIS*基因共转染至肺癌细胞系H460后，可有效提高H460的摄碘能力。

## Abstract (en)

**Background and objective:**

Lung cancer harms people's health or even lives severely. Especially, the therapy of non-small cell lung cancer (NSCLC) has not been obviously improved for many years. The aim of this study is to transfer the human sodium/iodide symporter (hNIS) and the human thyroperoxidase (hTPO) genes into H460 lung cancer cell line, and to study the uptake ability of iodide after co-transfected *hTPO* and *hNIS* gene in cell lines.

**Methods:**

Through cloning, recombination, packaging and amplifying, the recombinant adenosine virus (AdTPO) was constructed. Then the protein expression of AdTPO was tested by Western blot. After transfected *hNIS* gene into human lung cancer cell line H460 through liposome, stably expressing *hNIS* gene cell lines (hNIS-H460) selected by G418 antibiotics was determined as hNIS-H460 group. Using AdTPO, *hTPO* gene was transducted into hNIS-H460, as AdTPO-hNIS-H460 group. H460 cell without *hNIS* gene was applied as control group (H460). Then, we investigated the ^125^Ⅰ uptake assay of the above cells.

**Results:**

We were successful in co-transfecting *hNIS* and *hTPO* gene into human lung cell lines H460, and were obtained *hNIS* and *hTPO* gene lung cancer cell lines (hNIS-H460 and AdTPO-hNIS-H460). In AdTPO-hNIS-H460, hNIS-H460 and H460, the uptake ability of ^125^Ⅰ was (59 637.67±1 281.13), (48 622.17±2 242.28) and (1 440.17±372.86) counts·min^-1^. The uptake ability of ^125^Ⅰ was 41 fold higher in AdTPO-hNIS-H460 than in blank control H460 (*P* < 0.01), and 34 fold higher in hNIS-460 than in blank control H460 (*P* < 0.01), and 1.2 fold higher in AdTPO-hNIS-H460 than in hNIS-H460 (*P* < 0.01).

**Conclusion:**

The uptake ability of ^125^Ⅰ could increase by co-transfected *hNIS* and *hTPO* genes into human lung cancer cell lines H460.

肺癌是严重危害人类生命健康的常见病，目前国内外肺癌的发病率和死亡率还在不断上升。肺癌在城市占男性恶性肿瘤死亡的38.08%，女性的16%，均居首位。大多数肺癌患者确诊时已失去根治性手术切除的机会，全身化疗是一种有效的治疗。一项Ⅲ期随机试验结果^[1]^显示，化疗对晚期NSCLC的有效率为20%-40%，1年生存率为35%-45%，中位生存期也仅为8个月-9个月。NSCLC的治疗疗效有待进一步提高，并需要建立新型高效的治疗方法。

放射性碘是治疗恶性肿瘤的一种有效治疗手段，但其发挥杀伤肿瘤细胞效应的基础是肿瘤细胞必须具有摄取碘的能力，从而利用^131^Ⅰ发射的β射线发挥治疗作用。为了使原来不摄碘的肺癌细胞摄取碘，我们将人钠碘转运体(human sodium/iodide symporter, *hNIS*)基因引入大细胞肺癌细胞系H460；为了延长碘在甲状腺细胞中的滞留时间，使肿瘤细胞摄碘能力明显增高，我们同时共转染入人甲状腺过氧化物酶(human thyroperoxidase, *hTPO*)基因，从而达到提高治疗效果的目的。

## 1 材料与方法

### 1.1 材料和仪器

H460人大细胞肺癌细胞由中国医学科学院放射医学研究所惠赠，pcDNA3.1/hNIS质粒由本实验室构建并保存。G418硫酸盐、DMEM高糖型培养基和脂质体(Lipofectamine^TM^ 2000)购自美国Invitrogen公司；实验用酶类均购自日本Takara(宝生物)公司。^125^Ⅰ购自中国中核高通公司。1261型γ计数器为澳大利亚LKB公司生产。

### 1.2 实验方法

#### 1.2.1 重组腺病毒的构建

##### 1.2.1.1 hTPO的亚克隆

将hTPO插入腺病毒穿梭载体pAdTrack-CMV，C端融合6×His标签，插入位点为NotⅠ和HindⅢ，并对目的基因测序。

##### 1.2.1.2 获得腺病毒载体质粒AdTPO

使用限制性内切酶PmeⅠ线性化pAdTrack-CMV-TPO，并与腺病毒骨架质粒AdEasy-1共同转化重组菌感受态大肠杆菌BJ5183，获得重组质粒，*PacI*酶切鉴定电泳。

##### 1.2.1.3 重组腺病毒AdTPO的包装、扩增及纯化

293细胞培养，使用病毒质粒转染293细胞，以PacI线性化AdTPO质粒转染293细胞，转染7天后收集细胞反复冻融3次，离心获得P0代病毒，以一定量的P0代病毒感染新的293细胞收集并持续感染获得P1及P2代病毒。以腺病毒纯化试剂盒(CBLabs, Cat.No: VPK-100)纯化P2代病毒，并检测重组腺病毒滴度测定：滴度(VP/mL)=OD_260_×dilution×10^12^ VP/mL。

#### 1.2.2 Western blot检测目的基因重组腺病毒AdTPO的表达

pAdTrack-CMV-TPO质粒转染293细胞，并以空载体转染平行细胞作为阴性对照，48 h后裂解细胞并Western blot检测，检测抗体为His单抗(Genscript, Cat.No.: A00186)。

#### 1.2.3 脂质体法转染重组质粒pcDNA3.1-hNIS至H460细胞^[1]^

以SDS碱裂解法，提取质粒pcDNA3.1-hNIS。用Lipofectamine^TM^ 2000进行转染，G418硫酸盐筛选转染后的细胞，2周后出现抗G418的阳性克隆，即稳定表达hNIS基因的H460细胞系。

#### 1.2.4 在稳定表达hNIS的H460细胞系中实现重组腺病毒AdTPO的共转染

将稳定表达hNIS的H460细胞以1×10^8^/L接种于6孔板。待细胞长至80%-90%融合后，吸弃培养基，每孔加入含感染复数(MOI=100)的用无血清培养基稀释的病毒悬液5×10^4^/L，37 ℃、5%CO_2_孵箱培养。1 h后加入足量无血清培养液继续培养。6 h后吸去含病毒液培养基，加入低血清培养液(5%FBS)的完全培养基，继续在37 ℃、5%CO_2_培养箱中培养24 h。

#### 1.2.5 测定hNIS联合AdTPO共转染H4 60细胞后的摄^125^Ⅰ能力

将hNIS单独转染、hNIS联合AdTPO共转染的H460细胞以1×10^5^/孔的密度接种于6孔板，并设置对照组(H460)，每组重复6孔。每孔细胞均在原培养基中加入1850 Bq的^125^Ⅰ，在37 ℃、5%CO_2_培养箱中培养90 min后收集细胞，使用γ计数器测量细胞每分钟放射性计数cpm值。

#### 1.2.6 测定hNIS联合AdTPO共转染H460细胞后的^125^Ⅰ外流及有效半衰期

hNIS联合AdTPO共转染组(实验组)、H460单独转染组(阴性对照组)和未进行任何转染的空白对照组，在1 850 Bq的^125^Ⅰ环境中孵育120 min后，使用新的10%DMEM培养基置换含^125^Ⅰ的培养基，分别在0 min、5 min、10 min、15 min、20 min、25 min、30min测定细胞的放射性活性，绘制^125^Ⅰ外流曲线，并测定有效半衰期，公式见下。每组测定6孔细胞。

有效半衰期公式：

$
\frac{1}{{\rm{T}}} = \frac{1}{{{\rm{T'}}}} + \frac{1}{{{\rm{T''}}}}
$

生物半衰期是T'，物理半衰期为T"，有效半衰期为T。

#### 1.2.7 统计学处理

所得数据用方差分析进行比较，样本均数两两比较用*q*检验(*Newman-Keuls*法)，以*P* < 0.05为差异有统计学意义，采用SPSS 15.0系统进行统计学分析。

## 2 结果

### 2.1 重组腺病毒的构建

#### 2.1.1 hTPO的亚克隆

将目的*hTPO*基因使用NotⅠ和HindⅢ酶切位点，插入腺病毒穿梭载体pAdTrack-CMV。

#### 2.1.2 腺病毒载体质粒AdTPO的获得

限制性内切酶*PmeⅠ*线性化重组穿梭质粒pAdTrack-CMV-TPO并与腺病毒骨架质粒AdEasy-1共转化重组大肠杆菌BJ5183，然后提取重组病毒，并使用*PacⅠ*限制性内切酶，酶切重组病毒并电泳鉴定，显示可将病毒切成长约4 500 bp及18 000 bp左右的片段，与预期一致，结果见图 1。

**1:**
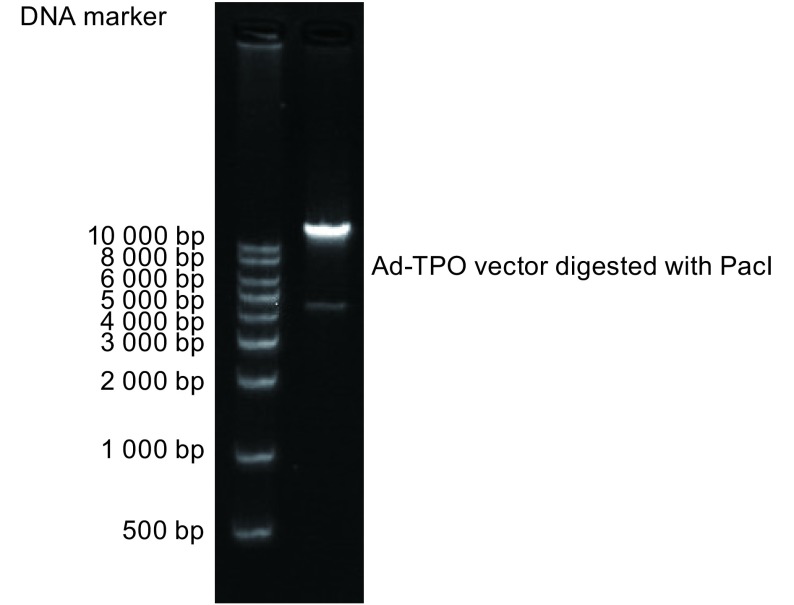
PacI酶切AdTPO载体 Ad-TPO vector digested with PacI

#### 2.1.3 包装、扩增及纯化AdTPO病毒

线性化的AdTPO质粒经脂质体包裹后转染293细胞，24 h后可见绿色荧光表达，7天-10天后荧光显微镜下可见大量细胞有绿色荧光，细胞变圆。由于初次收集的病毒滴度低，需要多次感染293细胞，在7天后收集293细胞反复冻融3次，离心获得P0代病毒，并持续感染获得P1及P2代病毒，以腺病毒纯化试剂盒(CBLabs, Cat.No: VPK-100)纯化P2代病毒，并检测OD_260_=0.255。滴度(VP/mL)=OD_260_×dilution×10^12^=0.205×5×10^12^=1.025×10^12^ VP/mL。

### 2.2 Western blot检测目的基因重组腺病毒AdTPO的表达

pAdTrack-CMV-TPO质粒转染293细胞，并以空载体转染平行细胞作为阴性对照，48 h后裂解细胞并Western blot检测，检测抗体为His单抗(Genscript, Cat.No: A00186)，Western blot显示90 kDa大小处有阳性反应条带出现，结果见图 2。

**2:**
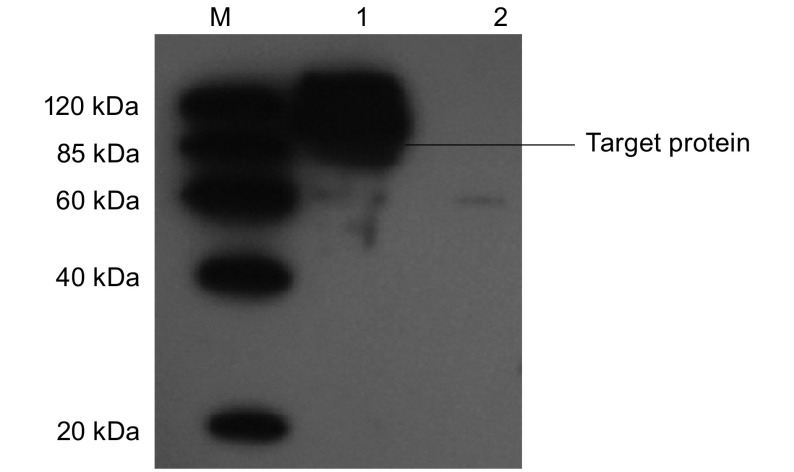
Western blot结果。M：Marker条带；1：pAdTrack-CMV-TPO转染后细胞系；2：对照组细胞系。 Result of Western blot. M: Marker; 1: lysate of cells transfected with pAdTrack-CMV-TPO; 2: lysate of control cells.

### 2.3 稳定表达*hNIS*基因的细胞系hNIS-H460的获得

使用脂质体转染法转染*hNIS*基因后，经浓度为600 ng/L的G418筛选后获得稳定表达*hNIS*基因的细胞系hNIS-H460。

### 2.4 重组腺病毒AdTPO的共转染

在稳定表达hNIS的H460细胞系中实现重组腺病毒AdTPO的共转染，获AdTPO-hNIS-H460细胞系。其病毒感染复数MOI=100。

### 2.5 hNIS联合AdTPO共转染H460细胞后的摄^125^Ⅰ能力

在1 850 Bq的^125^Ⅰ环境中孵育90 min后，AdTPO-hNIS-H460细胞每分钟放射性计数为59 637.67±1 281.13，hNIS-H460细胞为48 622.17±2 242.28，H460细胞为1 440.17±372.86。三组间总体具有统计学差异(*F*=576.38, *P* < 0.01)，AdTPOhNIS-H460组较对照组(H460)摄^125^Ⅰ能力增高约41倍(*t*=106.84, *P* < 0.01)，hNIS-H460组较对照组(H460)摄碘能力增高约34倍(*t*=50.84, *P* < 0.01)，AdTPO-hNIS-H460组较hNIS-H460组高约1.2倍(*t*=10.45, *P* < 0.01)。见图 3。

**3:**
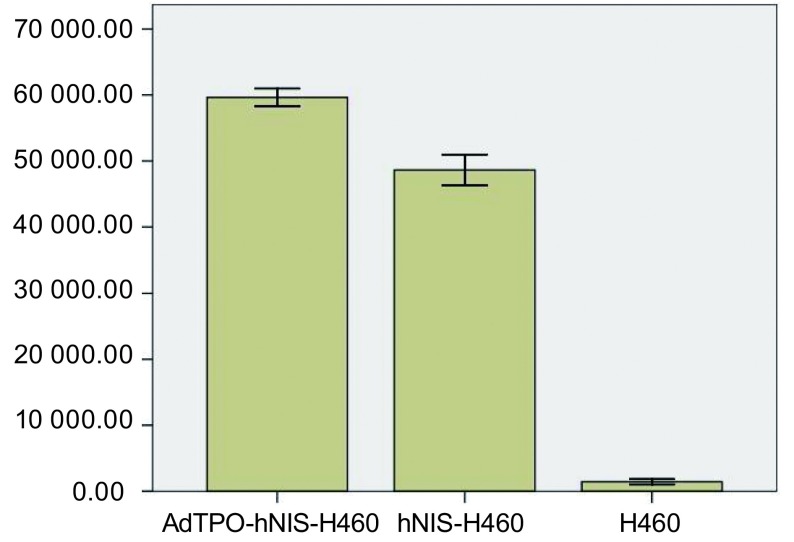
G418筛选后各细胞系的摄^125^Ⅰ能力 The ^125^Ⅰ uptake ability of cell lines after selected by G418 antibiotics

### 2.6 hNIS联合AdTPO共转染H460细胞后的^125^Ⅰ外流及有效半衰期测定

在1 850 Bq的^125^Ⅰ环境中孵育120 min后，分别在不同时间测定细胞的放射性活性，绘制AdTPO-hNISH460(实验组)、hNIS-H460(阴性对照组)和H460(空白对照组实验组)的外流曲线，结果如图 4。结果表明实验组AdTPO-hNIS-H460在环境中撤掉^125^Ⅰ后，细胞中的^125^Ⅰ外流速度较阴性对照组相比减慢，实验组AdTPO-hNIS-H460中^125^Ⅰ的有效半衰期为14 min，阴性对照组hNIS-H460中^125^Ⅰ的有效半衰期为7 min，空白对照组细胞H460因为不能摄取^125^Ⅰ，故其外流曲线不随时间变化，故可知hNIS与H460共转染后细胞内的碘储留时间比较单独转染hNIS的细胞碘储留时间延长。

**4:**
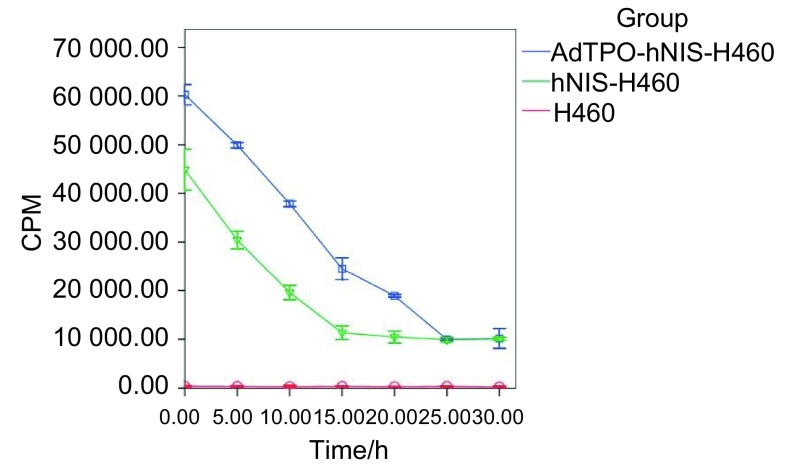
各组细胞^125^Ⅰ外流图 ^125^Ⅰ efflux experiments of cell lines

## 3 讨论

放射性碘用于甲状腺疾病的显像和治疗是一种非常成熟而有效的方法。在甲状腺中，碘是通过*hNIS*基因被主动转运入细胞的^[2, 3]^。如今，*hNIS*基因已经被广泛地转染至肿瘤细胞，如甲状腺癌、肺癌、乳腺癌、宫颈癌、卵巢癌、前列腺癌、肝癌、胰腺癌、结肠癌、神经胶质瘤、黑色素瘤、骨髓瘤等^[4-11]^，并成功地使这些肿瘤细胞具有摄碘功能。为了提高治疗效果，可以将hNIS联合*hTPO*基因共转染肿瘤细胞以延长碘在细胞中的停留时间；还可以将*NIS*基因改造成具有肿瘤特异性启动子的重组基因，使其具有肿瘤靶向性，避免损伤正常组织，增强^131^Ⅰ的杀伤力。

一些研究者将*NIS*基因转入不摄取碘的甲状腺肿瘤和非甲状腺肿瘤细胞，使其表达NIS蛋白而利用^131^Ⅰ治疗，且均成功地将*NIS*基因转入这些肿瘤中并表达出有功能的NIS蛋白。然而，由于碘从肿瘤细胞中流出太快^[12]^，^131^Ⅰ只能在较短的时间内对肿瘤组织进行破坏，从而达不到明显的治疗效果。同时，在转染率相对较低的情况下，虽然可在肿瘤细胞表达出有功能的NIS蛋白，但如果表达的NIS数量不足则可能会导致其摄取的^131^Ⅰ的量不足而不能起到治疗作用。另外，在这种情况下，^131^Ⅰ相对较短的半衰期(8天)和β粒子较低的能量可能也是导致该结果的原因之一。因此，为了克服这些问题，学者们应用了一些不同的方法，其中包括*hNIS*联合*hTPO*基因共转染肿瘤细胞以延长碘在细胞中的停留时间^[13]^。

甲状腺过氧化物酶(thyroid peroxidase, TPO)在正常甲状腺中参与碘的有机化过程，将进入甲状腺细胞内的Ⅰ^-^有机化，从而延长碘在甲状腺细胞中的滞留时间。正常甲状腺组织和甲状腺癌组织内碘的生物半衰期分别约为60天和10天，正是因为甲状腺内的TPO使碘有机化才可使碘在细胞内停留足够长的时间。因此，如果将*NIS*和*TPO*基因联合转染NIS表达阴性的肿瘤细胞，使其摄取碘并使得摄入细胞内的碘有机化，从而延长放射性碘在肿瘤细胞内的滞留时间，达到提高治疗效果的目的。Wenzel等^[14]^将*NIS*、*TPO*基因单独及联合转染甲状腺癌细胞，结果显示*TPO*基因单独转染组及*NIS*、*TPO*基因联合转染组可观察到碘的有机化，而NIS单独转染组未观察到碘的有机化。本研究使用脂质体法将*hNIS*基因转染至大细胞肺癌细胞系H460细胞中，通过G418筛选后成功获得了稳定表达*hNIS*基因的H460细胞系，并在稳定表达hNIS的H460细胞系中实现重组腺病毒AdTPO的共转染，获得AdTPO-hNIS-H460细胞系，进行稳定表达细胞系的体外摄^125^Ⅰ实验。结果显示AdTPO-hNIS-h460组与hNIS-H460组相比摄碘能力有所增高，证明*hNIS*和*hTPO*基因共转染可以增强H460细胞的摄碘能力。而且实验组AdTPO-hNIS-H460中^125^Ⅰ的有效半衰期比较对照组hNIS-H460的有效半衰期延长，证明AdTPO-hNIS-H460组中细胞碘滞留时间比hNIS-H460有所延长，但是延长的碘滞留时间是否能强化放射性碘对肿瘤细胞的杀伤效果，还需进一步研究。

2001年Huang等^[15]^首次报道将*NIS*、*TPO*基因克隆至含有巨细胞病毒启动子的质粒中并将该重组质粒转染到非小细胞肺癌中，结果发现肿瘤细胞对碘的摄取能力增加，碘在肿瘤细胞内的滞留时间显著延长，肿瘤细胞的凋亡也明显增加。我们成功将*NIS*及*TPO*基因共转染入神经胶质瘤细胞中并初步证明，其共转染可以延长胶质瘤细胞中碘的滞留时间，但是以前的研究采用的是质粒转染系统，只能做细胞水平的转染而不能用作动物体内研究，且前次TPO的转染为瞬时转染未筛选，转染效率低^[16]^。与之相比，本次研究使用腺病毒载体系统以携带*hTPO*基因，转染效率高，效果好，更能准确地体现细胞转染后摄碘情况。重组腺病毒可以有效地转染分裂和静止期细胞，而且携带基因不整合到宿主细胞基因组中，无潜在致癌危险^[17]^。本实验中采用的腺病毒载体AdEasy系统是近年来较新的载体系统，它重组效率高、周期短；利用含不同抗生素的平板筛选重组子，简化了筛选工作；经重组的腺病毒带有绿色荧光蛋白(green fluorescent protein, GFP)基因，可直接通过荧光显微镜监测病毒的生成，且GFP表达与空斑形成实验之间具有很好的一致性，结果可信。因此，应用该系统可方便地生成重组腺病毒，进一步拓宽了腺病毒在基因治疗中的应用。

理想的基因治疗方案必须具备有效性和安全性。在目前的研究中，组织特异性启动子的应用在提高治疗效果方面是一个重要的突破。有很多研究将*NIS*基因改造成具有肿瘤特异性启动子的重组基因，使其特异性地表达于肿瘤细胞，从而提高肿瘤细胞摄碘的数量，增强^131^Ⅰ的杀伤力。Chen等^[18]^应用白蛋白启动子和增强子调控的NIS逆转录病毒构建了稳定表达NIS的肝癌细胞系MHmAlbhNIS6，^131^Ⅰ治疗实验结果较满意。更值得一提的是，hTERT启动子作为一种广谱的肿瘤细胞的特异性启动子，可在转录水平调控目的基因的表达，以确保对肿瘤细胞的杀伤，而正常组织细胞则不受到伤害，为今后体内实验中如何保护正常组织不受误伤的研究确立了方向。随着各相关研究的进一步发展，基因治疗领域还将取得更新的进展。
